# LMAN1–MCFD2 complex is a cargo receptor for the ER-Golgi transport of α1-antitrypsin

**DOI:** 10.1042/BCJ20220055

**Published:** 2022-04-11

**Authors:** Yuan Zhang, Min Zhu, Chunlei Zheng, Wei Wei, Brian T. Emmer, Bin Zhang

**Affiliations:** 1 Genomic Medicine Institute, Lerner Research Institute of Cleveland Clinic, Cleveland, OH, U.SA.; 2 Department of Pathology, Karamay Central Hospital, Karamay, China; 3 Department of Internal Medicine and Life Sciences Institute, University of Michigan, Ann Arbor, MI, U.S.A.

**Keywords:** cargo proteins, cellular secretion, endoplasmic reticulum, intracellular transport, serpin

## Abstract

α1-antitrypsin (AAT) is a serine protease inhibitor synthesized in hepatocytes and protects the lung from damage by neutrophil elastase. AAT gene mutations result in AAT deficiency (AATD), which leads to lung and liver diseases. The AAT Z variant forms polymer within the endoplasmic reticulum (ER) of hepatocytes and results in reduction in AAT secretion and severe disease. Previous studies demonstrated a secretion defect of AAT in LMAN1 deficient cells, and mild decreases in AAT levels in male LMAN1 and MCFD2 deficient mice. LMAN1 is a transmembrane lectin that forms a complex with a small soluble protein MCFD2. The LMAN1–MCFD2 protein complex cycles between the ER and the Golgi. Here, we report that LMAN1 and MCFD2 knockout (KO) HepG2 and HEK293T cells display reduced AAT secretion and elevated intracellular AAT levels due to a delayed ER-to-Golgi transport of AAT. Secretion defects in KO cells were rescued by wild-type LMAN1 or MCFD2, but not by mutant proteins. Elimination of the second glycosylation site of AAT abolished LMAN1 dependent secretion. Co-immunoprecipitation experiment in MCFD2 KO cells suggested that AAT interaction with LMAN1 is independent of MCFD2. Furthermore, our results suggest that secretion of the Z variant, both monomers and polymers, is also LMAN1-dependent. Results provide direct evidence supporting that the LMAN1–MCFD2 complex is a cargo receptor for the ER-to-Golgi transport of AAT and that interactions of LMAN1 with an N-glycan of AAT is critical for this process. These results have implications in production of recombinant AAT and in developing treatments for AATD patients.

## Introduction

Glycoprotein α1-antitrypsin (AAT) is an abundant serine protease inhibitor in the blood. It is mainly produced by the liver, and is essential in preventing destruction of lung parenchyma by inhibiting the neutrophil elastase [1–3]. In addition, AAT is also an acute phase reactant and may have anti-inflammatory, immunomodulatory and antimicrobial properties [2,4,5]. In particular, AAT has shown promise in treating type 1 diabetes and enhancing islet engraftment [6–8]. AAT deficiency (AATD), which is characterized by reduction in serum AAT levels, is an autosomal codominant disease caused by AAT gene mutations, affecting ∼1 in 2000 to 1 in 5000 individuals [1,3]. Patients with AATD usually suffer from chronic obstructive pulmonary disease (COPD) and emphysema. Among all the disease-causing AAT variants, the Z variant (E342K) is relatively common and is most responsible for severe disease. The Z protein forms polymers and aggregates in hepatocytes, which not only results in lung disease, but also liver disease due to gain of toxic function. Another common AAT variant is the S variant (E264V). Although individuals with homozygous S variant do not have symptoms as the AAT levels appear sufficient, it causes disease when it is co-inherited with the Z variant or null variants [1,3].

Thorough understanding of the intracellular trafficking of AAT is important in understanding the disease mechanisms of AATD and designing new therapies for the disease. As other secreted proteins, AAT is transported to the extracellular space through the secretory pathway. Its folding is assisted by ER chaperones and monitored by the glycoprotein quality control system, including the calnexin/calreticulin cycle [9]. Unfolded and aggregated AAT molecules are degraded through the ER-associated degradation (ERAD) pathway, or through the autophagy pathway for aggregated proteins [9]. Properly folded cargo proteins are packaged into COPII (coat protein complex-II) vesicles for ER exit [10,11] and subsequently transported to the Golgi for further modification and sorting. Although ER export by bulk flow (passive diffusion into budding vesicles) might be sufficient for the transport of some proteins [12,13], many other proteins may need cargo receptors to promote their incorporation into COPII vesicles [14,15].

A previous study identified LMAN1, also called ERGIC-53, as a protein that interacts with AAT and potentially serves as a receptor for intracellular transport of AAT from the ER to the Golgi [16]. LMAN1 is a type I transmembrane protein with a carbohydrate binding domain (CRD) with homology to plant l-type lectin that binds mannose. It forms a Ca^2+^-dependent heterohexameric complex with MCFD2 in the ER lumen [17–19]. MCFD2 is a small soluble protein with two EF-hand domains [20]. Mutations in either gene cause a genetic bleeding disorder called combined deficiency of factor V (FV) and factor VIII (FVIII) (F5F8D) [21]. The LMAN1–MCFD2 complex cycles between the ER and the *cis*-Golgi, and acts as a cargo receptor for FV and FVIII, and potentially other proteins including AAT [16,22,23], Mac-2 binding protein (Mac-2BP) [24], cathepsin C [25], cathepsin Z [26], γ-aminobutyric acid type A receptors (GABAARs) [27] and matrix metalloproteinase-9 (MMP-9) [28]. The requirement for LMAN1–MCFD2 complex formation is a unique feature of this cargo receptor. However, MCFD2 appears dispensable for certain cargo proteins, such as cathepsin C and cathepsin Z [29]. We previously reported that LMAN1 and MCFD2 deficient mice exhibit liver accumulation of AAT, but only mild decreases in plasma AAT levels in male mice [23]. Although these results support a role for the LMAN1–MCFD2 complex in the ER-to-Golgi transport of AAT, other functions, including protein quality control, cannot be ruled out.

Here we characterized AAT secretion defects in LMAN1 and MCFD2 knockout (KO) human HepG2 and HEK293T cell lines, and demonstrated that ER-to-Golgi transport of AAT is delayed in both LMAN1 and MCFD2 KO cells, indicating that both LMAN1 and MCFD2 are required for the ER exit of AAT. The Z and S variants of AAT also depend on LMAN1 for efficient secretion. AAT secretion defects in LMAN1 KO cells were rescued by wild-type (WT) LMAN1, but not by LMAN1 mutants with carbohydrate binding deficiencies. Elimination of the N107 glycosylation site of AAT abolished LMAN1 dependent secretion. Our data provide direct evidence that the LMAN1–MCFD2 complex is required for the ER-to-Golgi transport of AAT and that interaction of the CRD of LMAN1 with N-glycan of AAT is critical for this process. These results have implications for both *in vitro* production of recombinant AAT for augmentation therapy and *in vivo* gene therapy treatment for AATD patients.

## Results

### AAT secretion levels are decreased in LMAN1 or MCFD2 knockout cells

**Figure 1.**
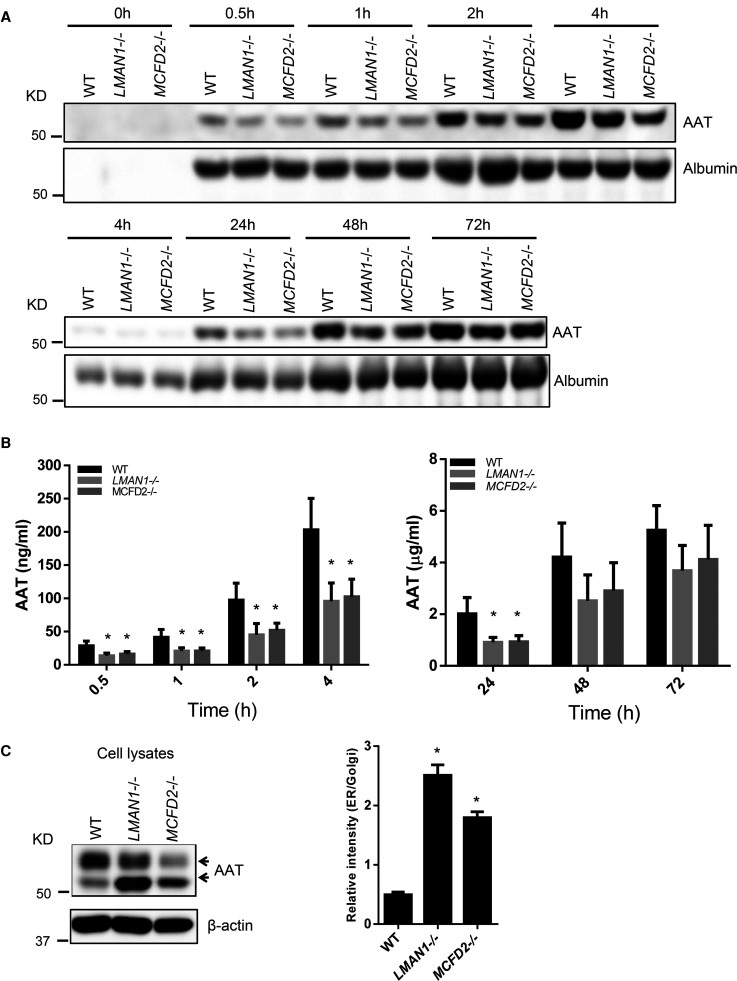
AAT secretion levels are decreased in LMAN1 and MCFD2 KO cells. (**A**) WT, LMAN1 KO and MCFD2 KO HepG2 cells were seeded in 60 mm plates, and a medium change was carried out the next day. At the indicated times after the medium change, conditioned media were collected and subjected to immunoblotting with anti-AAT and anti-albumin antibodies. (**B**) AAT concentrations in conditioned media were determined by a human AAT ELISA (data are mean ± SD, *n* = 3. * *P* < 0.05, statistical power >0.8). (**C**) Cells were collected 72 h after medium change. Cell lysates were analyzed by immunoblotting. Arrows indicate mature (upper band) and immature (lower band) fractions in cell lysates. Relative intracellular retention of AAT was expressed as ratios of ER (immature) and Golgi (mature) fractions. All KO cell data were compared with protein levels in WT cells (data are mean ± SD, *n* = 3. * *P* < 0.05).

We generated LMAN1 and MCFD2 KO HepG2 cell lines using the CRISPR–Cas9 system and verified the absence of target protein expression in clonal cell lines (Supplementary Figures S1, S2). Initial screening of two independent clones of KO cell lines showed that both clones had decreased AAT secretion (Supplementary Figure S3). One clone of each KO cell line was chosen for further studies. First, to study the detailed time course of endogenous AAT secretion in LMAN1 and MCFD2 KO cells, AAT levels in conditioned media of WT and KO cells were analyzed by immunoblotting (Figure 1A) and further quantified by ELISA (Figure 1B) at different time points after fresh medium exchange. During the first 4 h, the amount of AAT in conditioned media of LMAN1 or MCFD2 KO cells was ∼50% less than that of WT cells (Figure 1A,B). The discrepancy of secreted AAT between WT and KO cells persisted over longer time periods, but narrowed after culturing for 48–72 h (Figure 1A,B). There were no significant cell deaths between WT and KO cells during the 72 h incubation time. As a control for LMAN1-independent secretion, albumin levels were found to be unchanged between WT and KO cells (Figure 1A). Secretion defects were accompanied by higher intracellular AAT levels in both KO cell lines (Figure 1C). Intracellular AAT was detected as two bands by immunoblotting. The upper band is the mature AAT representing the Golgi fraction and the lower band is the immature AAT representing the ER fraction. The higher relative intensity of the lower band reflects AAT accumulation in the ER of the two KO cells, and suggests a defect in ER exit (Figure 1C).

We next isolated primary hepatocytes from WT, LMAN1 KO (*Lman1*^−/−^), MCFD2 KO (*Mcfd2*^−/−^) and DKO (*Lman1*^−/−^ *Mcfd2*^−/−^) mice [23]. AAT levels in conditioned media were monitored over a three day period by immunoblotting. At 24 h after medium change, AAT levels were markedly lower in conditioned media of cells derived from all three KO mice compared with WT cells. Similar to HepG2 cells, differences in the relative abundance of secreted AAT between WT and KO cells persisted, but narrowed over the next 48 h (Supplementary Figure S4). Levels of the immature fraction of intracellular AAT were higher in all three KO cells than in WT cells, whereas levels of the mature fraction were relatively lower in all KO cells (Supplementary Figure S4), also similar to HepG2 cells.

We next compared the intracellular localization patterns of AAT in primary hepatocytes. In WT hepatocytes, AAT is prominently detected in bright structures near nuclei, which co-localize with the *cis*-Golgi marker giantin (Supplementary Figure S5), although less prominent signal also co-localizes with the ER resident protein calreticulin. In all three KO hepatocytes, AAT staining patterns are distinct from the WT cells. Although Golgi staining is still readily detected, more AAT co-localizes with calreticulin in all three KO hepatocytes compared with WT cells (Supplementary Figure S5). These results are consistent with relative higher AAT Golgi fraction and lower ER fraction in WT cells than in KO cells (Figure 1C and Supplementary Figure S4).

### Rescue of AAT secretion defects in KO cells by re-introducing LMAN1 or MCFD2

**Figure 2.**
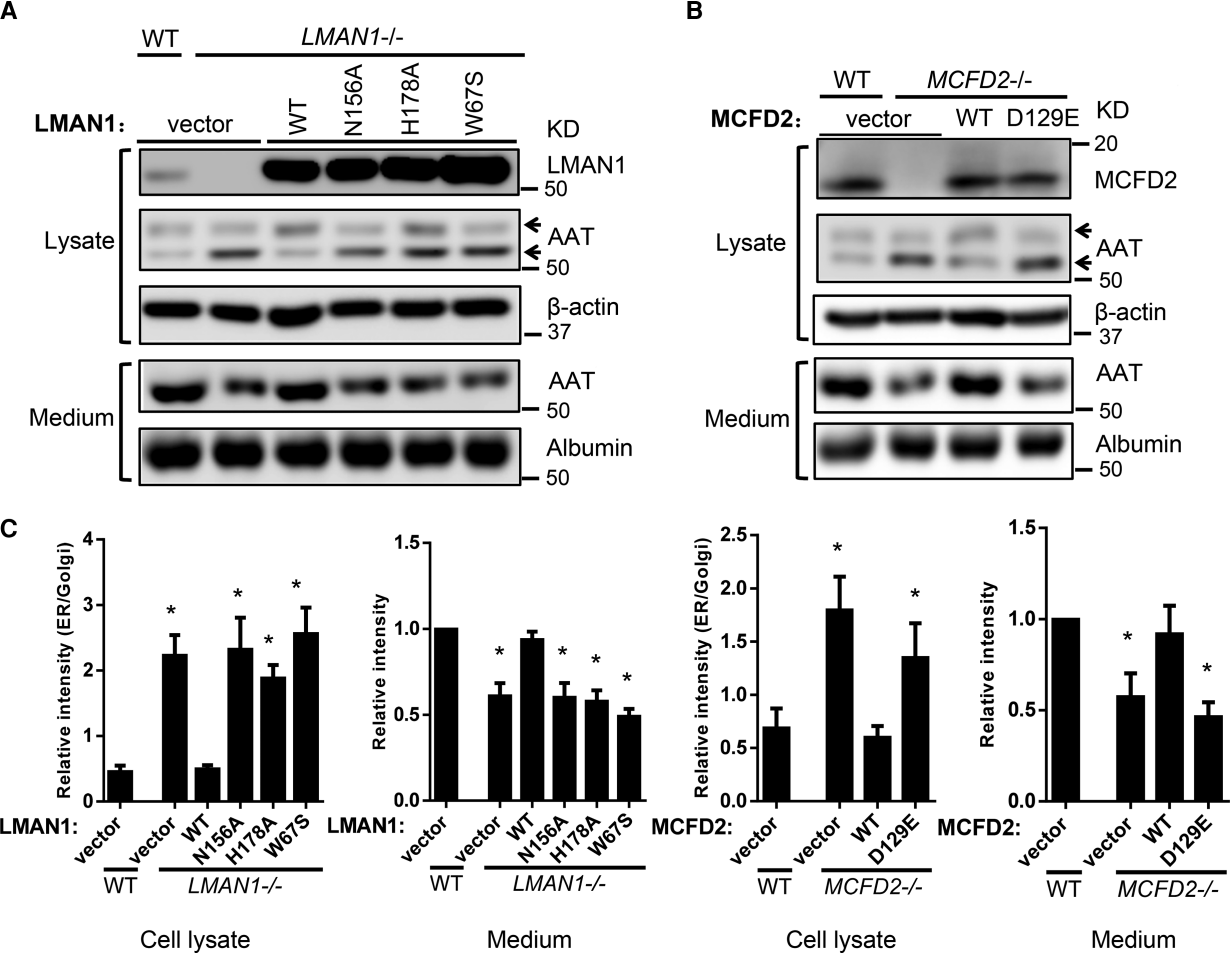
Rescue of AAT secretion defects in KO cells by re-introducing LMAN1 or MCFD2. WT LMAN1 and variants with indicated point mutations (**A**), as well as WT MCFD2 and the MCFD2^D129E^ mutant (**B**), were introduced into LMAN1 KO and MCFD2 KO HepG2 cells, respectively, by a retroviral vector. AAT expression in cell lysates and conditioned media were analyzed by immunoblotting. Arrows indicate mature and immature fractions in cell lysates. (**C**) Densitometry quantification of the immunoblotting data. Relative intracellular retention of AAT was expressed as ratios of ER (immature) fraction to Golgi (mature) fraction. AAT levels in media were expressed as fold changes to WT cells. All the data were compared with protein levels in WT cells (data are mean ± SD, *n* = 3. * *P* < 0.05).

Next, we introduced retroviral expression constructs into LMAN1 KO and MCFD2 KO HepG2 cells to stably express LMAN1 and MCFD2, respectively. Secreted AAT was analyzed by immunoblotting in media 4 h after fresh medium exchange and intracellular AAT was detected in cell lysates. After re-introducing LMAN1 or MCFD2 into KO cells, secreted AAT was restored to levels equivalent to that in WT cells (Figure 2). ER accumulation of AAT was also reversed, as measured by the relative intensity ratios of ER and Golgi bands (Figure 2). Expression of a patient-derived LMAN1 mutant (W67S) and mutants that abolish mannose binding (N156A and H178A) failed to rescue AAT secretion defects of LMAN1 KO cells, despite being expressed at levels much higher than the level in WT cells. Likewise, expression of a patient-derived MCFD2 missense mutant (D129E) failed to rescue AAT secretion defects in MCFD2 KO cells. These results indicate that the AAT secretion defects in LMAN1 or MCFD2 KO cells are due to the loss of LMAN1 or MCFD2 function.

### AAT is secreted at a lower rate in LMAN1 KO cells in cycloheximide chase experiments

**Figure 3.**
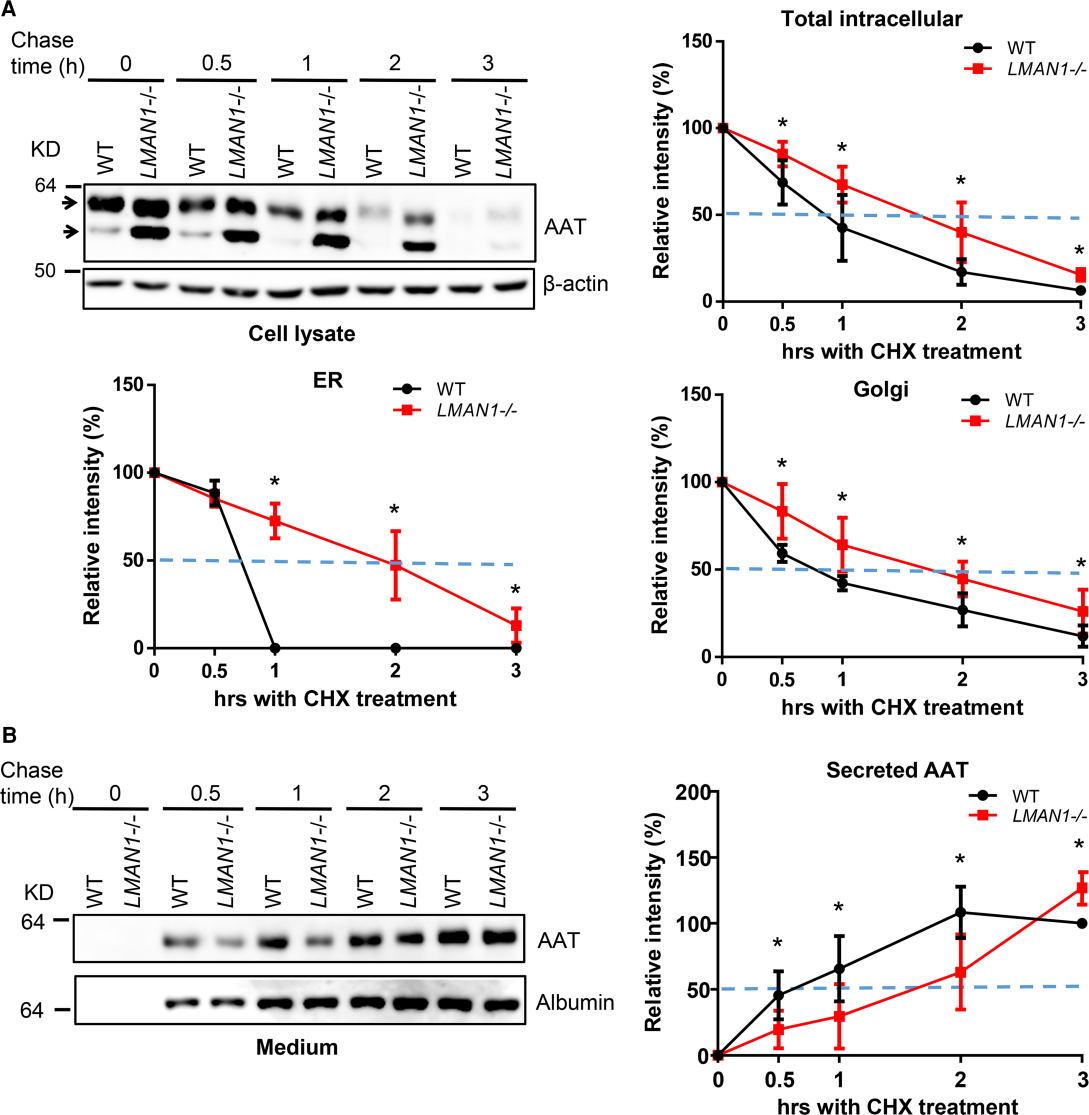
AAT is secreted at lower rate in LMAN1 KO cells in CHX chase experiments. WT and LMAN1 KO HepG2 cells were seeded in 24 well plates and treated with 200 µM CHX the next day. Samples were harvested at 0, 0.5, 1, 2 and 3 h. AAT levels in cell lysates (**A**) and conditioned media (**B**) were analyzed by immunoblotting. Relative protein levels during the CHX chase were quantified by measuring band intensities. Arrows indicate mature (Golgi) and immature (ER) fractions in cell lysates. Protein levels in cell lysates were plotted as fraction remaining, and protein levels in conditioned media were plotted as percentages of AAT levels in WT cells after 3 h CHX treatment. Dotted lines indicate 50% relative intensity (data are mean ± SD, *n* = 5. * *P* < 0.05, statistical power >0.8).

We performed cycloheximide (CHX) chase experiments to compare AAT secretion rates in WT and LMAN1 KO HepG2 cells. CHX inhibits protein translation, resulting in a decrease in the existing intracellular AAT protein levels over time (Figure 3A). We monitored levels of the mature and immature bands of intracellular AAT, as well as AAT secreted into conditioned media. At 1 h after CHX treatment, only ∼30% of total AAT remained inside WT cells, whereas ∼70% of total AAT remained inside LMAN1 KO cells (Figure 3A). Almost all the AAT protein had matured after 1 h CHX treatment in WT cells, but there was still a small amount of immature AAT remaining in LMAN1 KO cells after 3 h CHX treatment (Figure 3A). The pattern of the Golgi fraction is similar to the total AAT protein (Figure 3A). In contrast with intracellular protein levels, AAT levels remained significantly lower in conditioned media from KO cells compared with WT cells in the first 2 h after CHX treatment (Figure 3B). This is consistent with the faster decrease in intracellular AAT levels in WT cells, where little AAT was left inside cells compared with ∼40% AAT in LMAN1 KO cells at 2 h after chase. The remaining AAT in LMAN1 KO cells continued to secrete, reaching a level slightly higher than that from WT cells after 3 h treatment (Figure 3B). Our data suggest that the secretion rate of AAT is slower in LMAN1 KO cells than in WT cells.

### Analysis of ER to Golgi transport by the RUSH assay

**Figure 4.**
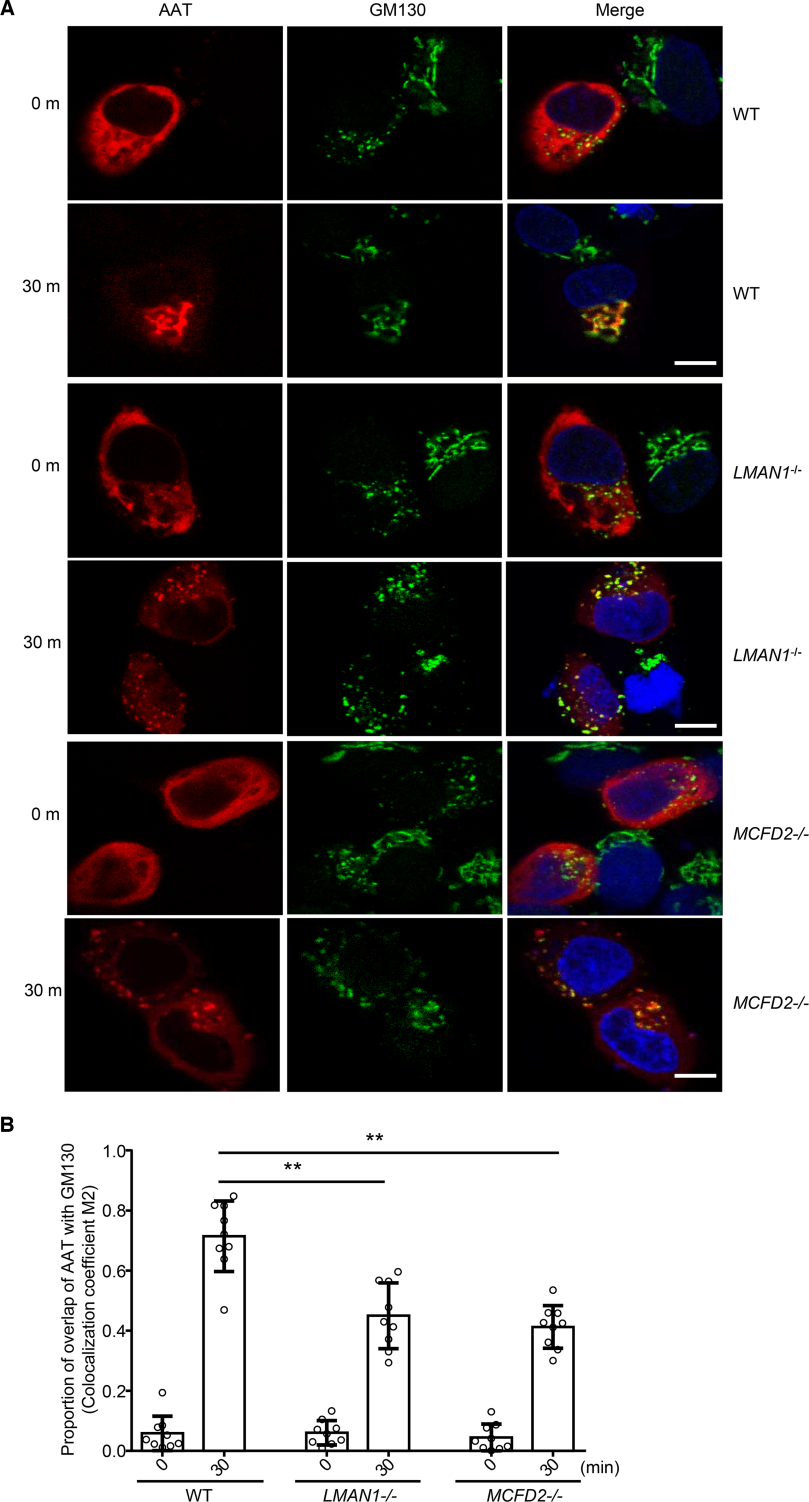
Analysis of ER to Golgi transport by the RUSH assay. (**A**) RUSH imaging of AAT in WT, LMAN1 KO and MCFD2 KO HepG2 cells. Images were taken before the addition of biotin (0 min) and at 30 m after biotin addition. GM130 was used as a Golgi marker. Scale bars, 10 µm. (**B**) Quantification of ER-to-Golgi traffic efficiency of AAT in WT and KO HepG2 cells. Colocalization of AAT with GM130 were quantified by colocalization coefficient M1. Each open circle represents one cell. (Data are mean ± SD, ** *P* < 0.01).

To directly compare the rate of ER-to-Golgi transport of AAT, we used the RUSH assay to analyze intracellular movement of the AAT-mCherry fusion protein in WT, LMAN1 KO and MCFD2 KO HepG2 cells. In this assay, cargo protein is first restricted to the ER by a streptavidin hook. The release from the hook is synchronized by the addition of biotin [30]. The localization of AAT-mCherry was monitored at different time points after biotin treatment. In WT cells, before the addition of biotin, AAT exhibited a typical ER staining pattern (Figure 4A). At 30 min after biotin addition, AAT had moved to the Golgi and co-localized with the *cis*-Golgi marker GM130 in WT cells. However, in LMAN1 KO cells, only a portion of AAT was co-localized with GM130 at 30 min after biotin addition, with the rest of AAT remaining in the ER (Figure 4A). Co-localized fractions of AAT with GM130 were quantified based on Mander's M2 coefficient [31]. Results showed significant delays in ER-to-Golgi transport of AAT in both LMAN1 KO and MCFD2 KO cells (Figure 4B).

### N-glycosylation site at N107 is required for LMAN1-dependent AAT secretion

**Figure 5.**
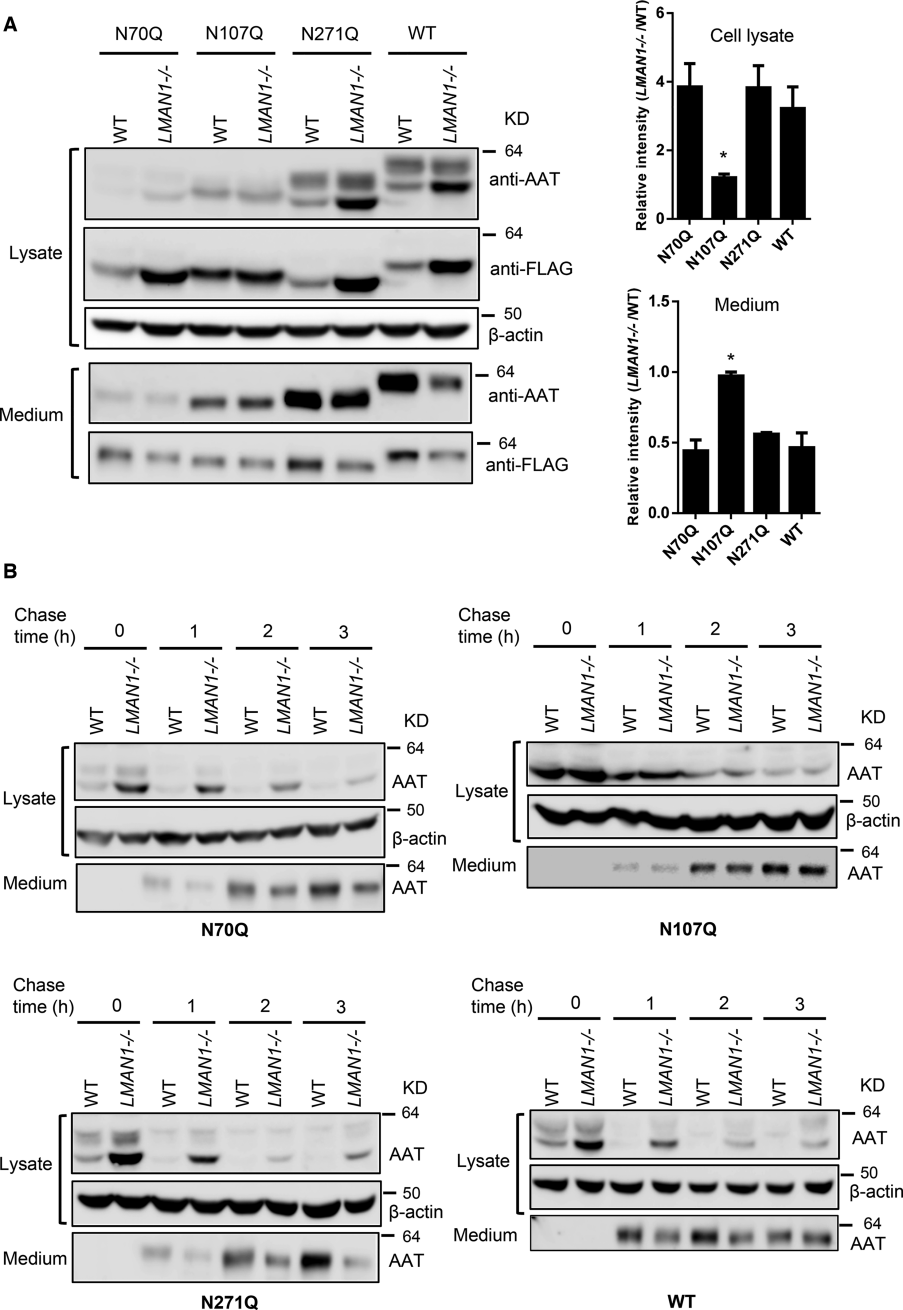
The N-glycan at N107 is important in LMAN1-dependent AAT secretion. (**A**) WT and LMAN1 KO 293T cells were transfected with FLAG-tagged AAT mutants. Conditioned media and cell lysates were collected 48 h after transfection and subjected to immunoblotting with anti-AAT or anti-FLAG antibody. Note that the anti-FLAG antibody was less efficient in detecting the Golgi fraction and the secreted AAT. Relative ER retention was expressed as ratios of the ER band intensities between lysates of LMAN1 KO and WT cells. Relative secretion was expressed as ratios of secreted AAT between conditioned media of LMAN1 KO and WT cells. All mutants were compared with WT AAT. Data are mean ± SD, *n* = 3. * *P* < 0.05. (**B**) Comparison of secretion rates of WT and N-glycosylation mutants of AAT with CHX-chase experiments. WT and LMAN1 KO 293T cells were transfected with FLAG-tagged AAT mutants. At 24 h post transfection, cells were treated with 200 µM CHX. Conditioned media and cell lysates were harvested at 0, 1, 2 and 3 h and AAT levels were analyzed by immunoblotting with anti-FLAG and anti-β actin antibodies.

As an animal lectin, LMAN1 presumably binds its glycoprotein cargo through interactions of the CRD with N-glycans. To identify N-glycans that mediate LMAN1-dependent AAT secretion, we engineered LMAN1 and MCFD2 KO 293T cell lines using the CRISPR–Cas9 system (Supplementary Figure S6). 293T cells were used because they lack endogenous AAT expression and have higher transfection efficiency than HepG2 cells. We individually mutated the three N-glycosylation sites of a C-terminally FLAG-tagged AAT, and transfected the resulting N70Q, N107Q, or N271Q mutant into WT and LMAN1 KO 293T cells. The intracellular and extracellular protein levels of the three single mutants were analyzed by immunoblotting. Both anti-AAT and anti-FLAG antibodies were used to detect AAT due to the weakened reactivity of N70Q and N107Q mutants to the anti-AAT antibody (Figure 5A). Both N70Q and N271Q mutants accumulated more intracellular protein in LMAN1 KO cells, and secreted less into conditioned media, which is similar to WT AAT. However, the N107Q mutant accumulated more intracellular protein in both WT and LMAN1 KO cells, with no additional accumulation observed in LMAN1 KO cells (Figure 5A). Similar levels of the N107Q mutant were detected in conditioned media of both WT and LMAN1 KO cells, indicating that the N107Q mutant secretion is not LMAN1-dependent. This effect was further analyzed by CHX chase experiments. Similar to WT AAT, N70Q and N271Q mutants were secreted faster in WT cells than in LMAN1 KO cells (Figure 5B). However, the N107Q mutant had a similar delayed secretion rate in both WT and LMAN1 KO cells (Figure 5B). Thus, the N-glycan at N107 plays an important role in LMAN1-dependent AAT secretion.

### Secretion of AAT Z or S variant is also partially dependent on LMAN1

**Figure 6.**
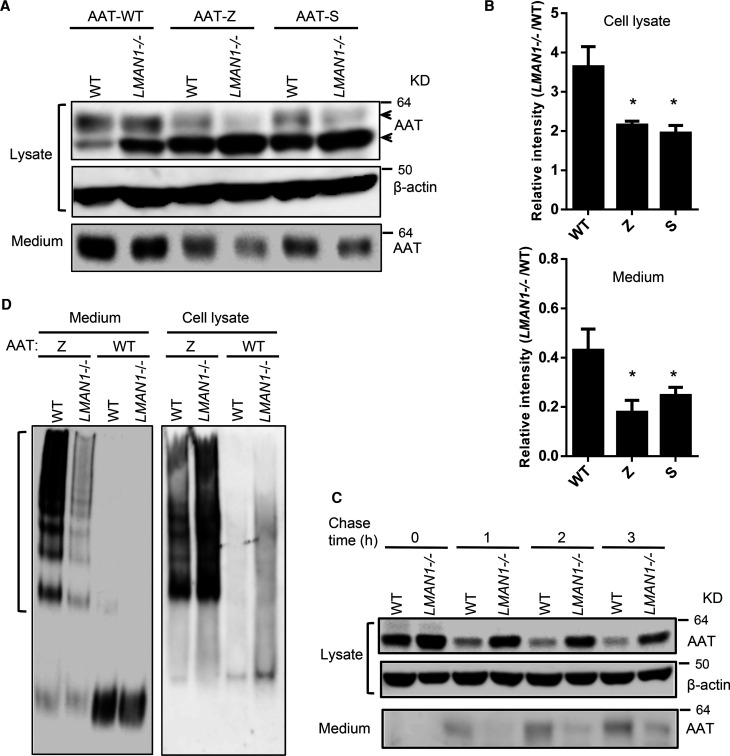
Analysis of secretion of AAT Z and S variants. (**A**) WT and LMAN1 KO 293T cells were transfected with FLAG-tagged AAT variants. Conditioned media and cell lysates were collected 48 h after transfection and subjected to immunoblotting with an anti-AAT antibody. (**B**) ER fractions in lysates and secreted AAT in media were quantified by densitometry. Relative ER retention was expressed as ratios of the ER band intensities between lysates of LMAN1 KO and WT cells. Relative secretion was expressed as ratios of secreted AAT between conditioned media of LMAN1 KO and WT cells. Data are mean ± SD, *n* = 3. * *P* < 0.05. (**C**) WT and LMAN1 KO 293T cells were transfected with FLAG-tagged AAT Z mutant. At 24 h post transfection, cells were treated with 200 µM CHX. Cell lysates and conditioned media were harvested at indicated times and AAT levels were analyzed by immunoblotting an anti-AAT antibody. (**D**) WT and LMAN1 KO 293T cells were transfected with FLAG-tagged AAT Z mutant and WT AAT. Conditioned media and cell lysates were collected 48 h post transfection and subjected to non-denaturing PAGE and immunoblotting with an anti-FLAG antibody.

Next, we analyzed the secretion of AAT Z (E342K) and S (E264V) variants in WT and LMAN1 KO cells. Intracellular levels of Z and S variants were higher than WT AAT in WT cells, consistent with the known secretion defects of Z and S variants (Figure 6A). Similar to WT AAT, which had an increase in intracellular level of the immature fraction in LMAN1 KO cells, Z and S variants also had further increases in intracellular levels of the immature fraction in LMAN1 KO cells (Figure 6B). Increased immature fractions were accompanied by decreased levels of mature fractions (Figure 6A). Conversely, overall secretion levels of Z and S variants were lower than that of WT AAT, and secretion was further decreased in LMAN1 KO cells for all three variants (Figure 6A,B). CHX chase experiment also showed that secretion of the Z variant was delayed in LMAN1 KO cells (Figure 6C).

The misfolded Z variant usually forms polymers and aggregates in the ER, resulting in reduction in secretion. To determine whether LMAN1 plays a role in AAT quality control and limits polymer secretion from the cell, we analyzed secreted WT and Z proteins by the native PAGE. In cell lysates, the Z variant was detected primarily as polymers, whereas WT AAT was mainly detected as a monomer (Figure 6D). The primary secreted form of the Z variant from WT cells was polymers, whereas WT AAT was mainly secreted as a monomer, consistent with a previous study of secreted Z variant in 16HBE cells [32]. Intriguingly, in LMAN1 KO cells, polymers were also the main secreted form of the Z variant. These results demonstrate that polymers are secreted in WT cells, as well as at a reduced level in LMAN1 KO cells.

### AAT binding to LMAN1 is independent of MCFD2

**Figure 7.**
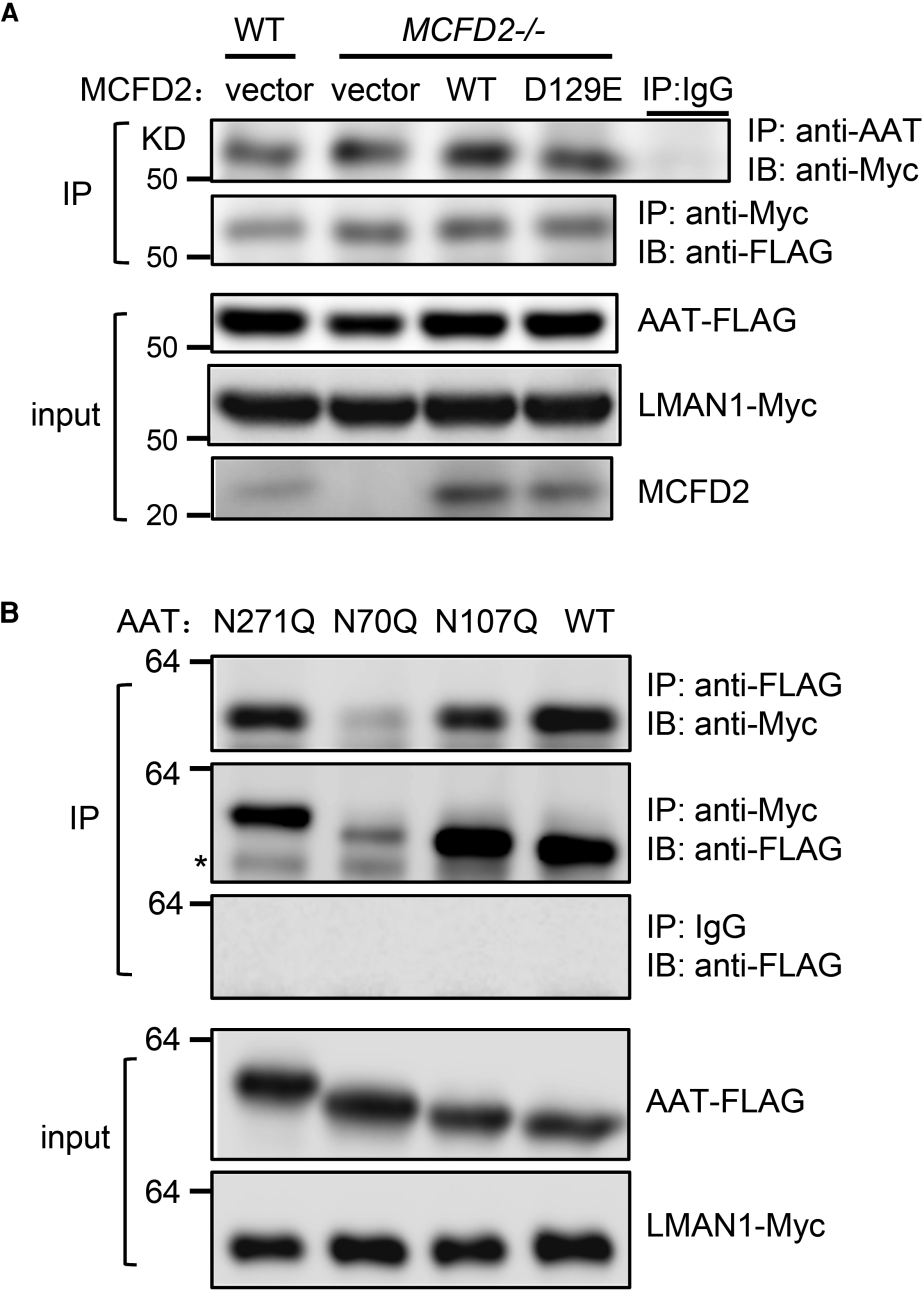
Co-immunoprecipitation of LMAN1 and AAT is independent of MCFD2. (**A**) Co-IP of FLAG-tagged AAT with Myc-tagged LMAN1 in WT and MCFD2 KO 293T cells, as well as in MCFD2 KO cells stably expressing WT MCFD2 and the MCFD2^D129E^ mutant. Cell lysates were immunoprecipitated with an anti-Myc antibody for LMAN1 and an anti-AAT antibody for AAT. Immunoprecipitates were subjected to immunoblotting with the indicated antibodies. Levels of LMAN1, AAT and MCFD2 in cell lysates (input) were also detected by immunoblotting. (**B**) FLAG-tagged AAT with Myc-tagged LMAN1 were co-transfected into 293T cells. Cell lysates were immunoprecipitated with an anti-FLAG antibody for AAT, an anti-Myc antibody for LMAN1 and murine IgG as a negative control. Immunoprecipitates were subjected to immunoblotting with the indicated antibodies. Asterisk denotes a nonspecific band. Levels of LMAN1 and AAT in cell lysates (input) were also detected by immunoblotting.

To validate the interaction of AAT and LMAN1, we co-expressed FLAG-tagged AAT and Myc-tagged LMAN1 in WT and MCFD2 KO cells. Cells were lysed in the presence of 2 mM CaCl_2_ and lysates were subjected to co-immunoprecipitation (co-IP) experiments with anti-AAT or anti-Myc antibody. In both WT and MCFD2 KO cells, anti-AAT antibody could pull down LMAN1 and anti-Myc antibody could pull down AAT, suggesting that AAT and LMAN1 can interact with each other in the absence of MCFD2 (Figure 7). We repeated this experiment in MCFD2 KO cells stably expressing WT MCFD2 or the MCFD2^D129E^ mutant and found that AAT could co-immunoprecipitate with LMAN1 to the same degree in MCFD2 KO cells as in WT cells (Figure 7). No co-IP was observed when non-specific IgG was used. These results suggest that AAT and LMAN1 binding is independent of MCFD2. To evaluate the importance of N-glycans of AAT in LMAN1 interaction, we performed co-IP experiments of WT and the three glycosylation mutants of AAT (Figure 5) using either anti-Myc or anti-FLAG antibody. Results showed that only the N107Q mutant had reduced co-IP with LMAN1, further supporting the importance of the N107 glycan in LMAN1 interaction (Figure 7B).

## Discussion

Previous studies have shown data that are consistent with LMAN1 serving as an intracellular transport receptor for AAT and identified ER accumulation of AAT in LMAN1, MCFD2 KO and double KO (DKO) mice, as well as mild decreases in plasma AAT in male mice of all three genotypes [16,22,23]. However, the precise role of LMAN1 and MCFD2 in intracellular transport of AAT was still unclear. In our current study, we compared detailed time course of endogenous AAT secretion in WT, LMAN1 and MCFD2 KO HepG2 cells and observed decreases in AAT secretion from both KO cells. Intracellular AAT accumulated in the ER of KO cells, which is consistent with our previous studies in LMAN1 and MCFD2 KO mouse liver [22,23]. We also obtained similar results with primary hepatocytes isolated from LMAN1 and MCFD2 single-KO and DKO mice. Rates of secretion and ER-to-Golgi transport were further assessed with CHX-chase and RUSH experiments. These analyses firmly established secretion defects of AAT due to delayed ER exit in both LMAN1 and MCFD2 KO cells. The observations that differences in steady-state levels of AAT between WT and KO cells became less profound 72 h after fresh medium change are in keeping with the *in vivo* data in mice. AAT levels were not drastically decreased in plasma of LMAN1 and MCFD2 KO mice despite the markedly increased accumulation of AAT in the ER of hepatocytes [22,23].

Our results indicate that LMAN1 and MCFD2 are both required for efficient ER-to-Golgi transport of AAT. WT MCFD2, but not a MCFD2 mutant defective in LMAN1 binding, was able to rescue the AAT secretion defect of MCFD2 KO cells. Thus, AAT belongs to the group of cargo proteins that LMAN1–MCFD2 complex formation is essential for cargo receptor function. This group also includes FV and FVIII, as mutations of either LMAN1 or MCFD2 result in F5F8D [21], and is different from a second group of cargo proteins that include cathepsins C and Z, which do not appear to require MCFD2 [29]. The observation that ER-to-Golgi transport of AAT was not completely blocked in the absence of LMAN1 or MCFD2 suggests the existence of an alternative transport receptor or transport through bulk flow that accounts for slower rates of ER-to-Golgi transport of AAT. Mild decreases in AAT levels in LMAN1 and MCFD2 deficient male mice are insufficient to cause AATD symptoms such as COPD and emphysema [23]. Human F5F8D patients are also not known to present AATD related symptoms [21].

LMAN1 appears to have all the features of a cargo receptor, yet MCFD2 is essential for the transport of at least a group of cargo proteins. What kind of role that MCFD2 plays in cargo transport is still unclear. Our previous studies showed that MCFD2 interacts with FV and FVIII independent of LMAN1 and EF-hand domains of MCFD2 mediate this interaction [20]. In this study, we detected co-IP of LMAN1 and AAT in the presence or absence of MCFD2, suggesting that LMAN1 can also interact with AAT independent of MCFD2. However, we cannot rule out the possibility that the affinity between LMAN1 and AAT is reduced in MCFD2 KO cells since co-IP is not a quantitative assay. Kawasaki et al. showed that MCFD2 enhanced the binding of the CRD of LMAN1 to the surface glycans of HeLa cells [33]. However, structural analyses showed that MCFD2 undergoes significant conformational alterations upon binding to LMAN1, and MCFD2 may operate as a recruitment factor for FV and FVIII [34,35]. These results suggest that AAT contains dual sorting signals that are recognized sequentially by MCFD2 and LMAN1.

Proper glycosylation is important for AAT secretion. Blocking N-linked glycosylation with tunicamycin dramatically decreased AAT secretion [36]. Mutations abolishing carbohydrate binding of LMAN1 failed to rescue the AAT secretion defect of LMAN1 KO cells, suggesting that N-glycans of AAT may serve as part of the recognition motif for LMAN1. However, eliminating different N-glycosylation site had different effects on secretion and intracellular accumulation of AAT. The N70Q and N271Q mutants secreted at comparable rates as WT AAT in WT cells, and had reduced secretion in LMAN1 KO cells similar to WT AAT, suggesting that elimination of these two glycosylation sites does not disrupt LMAN1-dependent AAT secretion. Thus, glycans at N70 and N271 sites likely do not play a major role in cargo recognition by LMAN1. On the other hand, the N107Q mutation reduced AAT secretion in both WT and LMAN1 KO cells with similar kinetics, highlighting the importance of N-glycan at N107 in LMAN1-dependent secretion. Co-IP experiment suggests that the N107Q mutant has markedly lower binding affinity for LMAN1. Previous studies suggested that glycosylation is not required for proper folding of AAT, and aggregation of unglycosylated protein appears to be a function of time spent in the unfolded state [36,37]. The N-glycan at N107 is either part of the cargo exit signal recognized by the LMAN1–MCFD2 complex, or elimination of this N-glycan leads to conformational changes to AAT structure that abolishes the exit signal.

A surprising finding is that Z and S protein transport is also LMAN1-dependent, as more mutant proteins accumulated intracellularly and secretion decreased in LMAN1 KO cells. A recent study using CHO-K1 cells also found a secretion defect of both monomeric AAT and a polymeric King's variant of AAT (H334D) in LMAN1-disrupted cells [38]. LMAN1-dependent Z, King's or S variant secretion suggests these mutations do not change the structure of the LMAN1-interacting motif, or the binding surface of AAT with LMAN1 may be located remotely from the mutation sites [39,40]. LMAN1 has previously been shown to play a role in quality control in the early secretory pathway [41,42]. However, we show that both monomeric and misfolded polymeric forms of the Z variant are secreted in both WT and LMAN1 KO cells, suggesting that LMAN1 is not a major quality control factor in AAT Z mutant secretion. Compounds that promote the interaction of LMAN1 and the Z variant may be helpful in relieving the ER aggregation of AAT polymers in liver of AATD patients.

AAT is an acute phase reactant and its acute phase induction may be important in the stress response, anti-inflammatory, immunomodulatory and antimicrobial functions of AAT [43–48]. Given the delayed secretion due to slower ER exit of AAT in LMAN1 and MCFD2 KO cells, it is possible that acute phase induction of AAT secretion is blunted in F5F8D patients. In the future, it will be interesting to study whether this is the case and what consequences may result. Recently, AAT was proposed to inhibit coronavirus entry by inhibiting the TMPRSS2 protease [49], and AATD patients may be more susceptible to severe COVID-19 [50]. It would thus be interesting to examine whether the SARS-CoV-2 virus can induce increased local secretion of AAT by neutrophils through acute phase induction, thus limiting the infection of SARS-CoV-2 in normal individuals, and whether this process is blunted in AATD and F5F8D patients.

## Methods

### Plasmid construction

The AAT cDNA was amplified from HepG2 cells by RT-PCR, and cloned into pcDNA3.1. A FLAG tag was engineered into the C-terminal end of the AAT sequence. Missense mutations were introduced into the construct using the QuickChange site-directed mutagenesis II XL kit (Agilent) and confirmed by Sanger sequencing. The pED-Myc-LMAN1 plasmid was derived from pED-FLAG-LMAN1 [51] by replacing the FLAG tag with the Myc tag using the PCR method.

### Reagents

Polyclonal antibodies against FLAG and c-Myc, as well as monoclonal antibodies against FLAG were purchased from Sigma–Aldrich (St. Louis, MO). Monoclonal anti-Myc antibody was purchased from Santa Cruz Biotechnology (Dallas, TX). Monoclonal anti-human AAT was from ProteinTech Inc (Rosemont, IL). Chicken anti-mouse AAT was from the Immunology Consultants Laboratory (Portland, OR). Cycloheximide (CHX) was obtained from Sigma–Aldrich. Protein A/G Plus-agarose beads were purchased from Santa Cruz Biotechnology.

### Cell culture

HepG2 cells were grown in ATCC-formulated Eagle's Minimum Essential Medium (EMEM) supplemented with 10% FBS, 100 IU/ml penicillin and 100 IU/ml streptomycin at 37°C and in 5% CO_2_. Human embryonic kidney 293T cells were grown in Dulbecco's Modified Eagle Medium (DMEM) supplemented with 10% FBS, 100 IU/ml penicillin and 100 IU/ml streptomycin at 37°C and in 5% CO_2_. Cells were transfected using FuGENE 6 (Promega, Madison, WI) according to the manufacturer's instructions. To control for variation in transfection efficiencies, experiments were independently performed 2–3 times. For CHX chase assays, cell media were replaced with prewarmed fresh media containing 200 µM CHX at 24 h after seeding for endogenous AAT or 24 h post transfection for transfected AAT. Cells and conditioned media were harvested at different time points for analysis.

### Establishment of LMAN1 and MCFD2 KO cells using the CRISPR–Cas9 system

Optimal *LMAN1* or *MCFD2* guide RNA (gRNA) sequences were selected by the Surveyor assay [52] and inserted into the CRISPR plasmid PX458 with a GFP marker (Addgene). The gRNA sequence for LMAN1 deletion is GGATCAGCTGATCTGTGGAA, and for MCFD2 deletion is CATCACTCATGTCCATAAGG. The KO cells were generated according to a protocol from Feng Zhang lab [53]. Briefly, individual PX458 plasmid with *LMAN1* or *MCFD2* sgRNA sequence inserted was transfected into 293T and HepG2 cells using lipofectamine 3000 (ThermoFisher, Waltham, MA). At 48 h post transfection, GFP positive cells were separated by fluorescence-activated cell sorting (FACS) with BD FACSAria II cell sorter (BD Biosciences, San Jose, CA). Clonal cell lines were derived by diluting cell suspensions to a single cell per well and expanding individual wells. Genomic DNA was isolated from clonal cell lines by QuickExtract (Lucigen, Middleton, WI) and target-site sequence was amplified by PCR and subjected to Sanger sequencing for genotyping with deconvolution of individual alleles using TIDE (Tracking of Indels by DEcomposition) [54]. Absence of target protein expression was confirmed by immunoblotting with anti-LMAN1 or anti-MCFD2 antibodies.

### Generation of cell lines stably expressing LMAN1 and MCFD2

To generate HepG2 and 293T stable cell lines, coding sequences of tagged LMAN1 and MCFD2 cDNA were amplified from pED-Flag-LMAN1 [19] or pcDNA3.1-MCFD2 [17] and cloned into the retroviral vector pMSCV [55]. WT plasmid was mutagenized for missense mutations using the QuikChange II XL Site-Directed Mutagenesis Kit. All expression constructs were validated by Sanger sequencing prior to transduction. Retroviruses were produced by co-transfecting retroviral vectors together with pVSV-G and gag/pol in a ratio of 2 : 1 : 2 into 293T cells using FuGENE 6. Media containing retrovirus were collected 48 h later and filtered through a 0.45 mm filter. Cells were transduced by retrovirus for 48 h in the presence of 8 µg/ml polybrene and kept under 2 µg/ml selection for 10 days prior to downstream experiments. Stable expression cell lines were maintained in 1 µg/ml puromycin thereafter.

### ELISA analysis of AAT levels in cell media

AAT concentrations in conditioned media were measured by a human AAT ELISA kit (R&D Systems, Minneapolis, MN) according to the manufacturer's instructions.

### Isolation and culture of primary mouse hepatocytes

Mouse hepatocytes were isolated from 6-month-old male WT, LMAN1 KO and LMAN1/MCFD2 DKO mice by the collagenase perfusion technique [56]. Mice were first anesthetized with pentobarbital (50 mg/kg) and perfused with the Hanks’ Balanced Salt Solution through the portal vein. Perfusate was allowed to drain to waste from the inferior vena cava. After the liver was perfused completely, the digestion medium (low glucose DMEM, 15 mM HEPES, 1.8 mM CaCl_2_, 100 units/ml collagenase) was perfused through portal vein until the liver was well digested. Hepatocytes were pelleted by centrifugation, assessed for viability by Trypan blue exclusion, which typically ranged from 70% to 90%, and cultured in high glucose DMEM containing 10% fetal bovine serum. The medium was changed to serum free low glucose DMEM after 4 h. Cell lysate and conditioned media were prepared at different time points. Animal experiments were carried out at Cleveland Clinic Lerner Research Institute and the experimental protocols were approved by the institutional animal care and use committee of Cleveland Clinic. Mice with unwanted genotypes from experimental breeding were killed by CO_2_ asphyxiation.

### Immunofluorescence imaging

Primary hepatocytes collected as above were grown on cover slips in serum free low glucose DMEM overnight, fixed in 4% paraformaldehyde, permeabilized with 0.1% Triton X-100, and incubated with either chicken anti-mouse AAT (ICL Lab, Portland, OR) followed by an Alexa Fluor 488-conjugated secondary antibody (ThermoFisher, Waltham, MA), or rabbit anti-calreticulin antibody or rabbit anti-giantin antibody (Sigma–Aldrich, St. Louis, MO) followed by an Alexa Fluor 596-conjugated secondary antibody. Cover slips were mounted on mounting media (Vector Lab, Burlingame, CA) and images were captured on an Olympus FluoView Confocal microscope.

### Immunoblotting and immunoprecipitation

Cells were lysed in Nonidet P-40 buffer containing CaCl_2_ (50 mM Tris–HCl, pH 7.5, 150 mM NaCl, 1% Nonidet P-40, 0.05% SDS, 2 mM CaCl_2_). Immunoblotting and immunoprecipitation experiments were performed as previously described [17–19]. Proteins in cell lysates and immunoprecipitates were separated in a 10% SDS–PAGE gel and subject to immunoblotting analysis with appropriate primary antibodies.

### RUSH (retention under select hook) assay

For RUSH experiments [30], the AAT coding sequence was amplified by PCR and inserted into plasmid li-Str_ST-SBP-mCherry (Addgene # 65269) through the SbfI site. WT, LMAN1 KO and MCFD2 KO HepG2 cells were seeded onto 12 mm glass coverslips one day before transfection. Plasmids were transfected into the cells using Lipofectamine 3000. After 24 h, media were changed into EMEM with 40 µM biotin to start the ER exit of AAT-mCherry. After various incubation time, cells were fixed with 4% paraformaldehyde, and permeabilized with 0.1% Triton X-100. After blocking with 3% bovine serum albumin, cells were incubated with a polyclonal rabbit anti-GM130 antibody (11308-1-AP, Proteintech, Rosemont, IL) followed by Alexa Fluor 488-conjugated anti-rabbit IgG antibody (ThermoFisher, Waltham, MA). Images were obtained by using a Leica confocal microscope (Wetzlar, Germany). For quantification of the co-localization of AAT and GM130, the images were analyzed by calculating Manders’ coefficient (M2) using the Volocity (Quorum Technologies, Ontario, Canada) software.

### Statistical analysis

All data are presented as mean ± standard deviation (SD). Statistical significance was calculated using two-tailed Student's *t*-test. *P*-values <0.05 were considered significant for all assays. Post hoc power calculation was performed using the G*Power program (Heinrich Heine University Düsseldorf, Germany).

## Data Availability

All data generated from this work are contained in the manuscript and its Supplementary Files.

## Open Access

Open access for this article was enabled by the participation of Cleveland Clinic in an all-inclusive *Read & Publish* agreement with Portland Press and the Biochemical Society under a transformative agreement with USA.

## Abbreviations

AAT: α1-antitrypsin
AATD: AAT deficiency
CHX: cycloheximide
COPD: chronic obstructive pulmonary disease
CRD: carbohydrate binding domain
DMEM: Dulbecco's Modified Eagle Medium
EMEM: Eagle's Minimum Essential Medium
ER: endoplasmic reticulum
FV: factor V
KO: knockout
SD: standard deviation
WT: wild-type
