# Supplementary material for: LMAN1–MCFD2 complex is a cargo receptor for the ER-Golgi transport of α1-antitrypsin
Source: Biochem J. 2022 Apr 11;479(7):839–55. doi: 10.1042/BCJ20220055 (PMC9022998; doi:10.1042/BCJ20220055)
Supplement: Supplementary Material [file BCJ-479-839-s1.pdf]

A

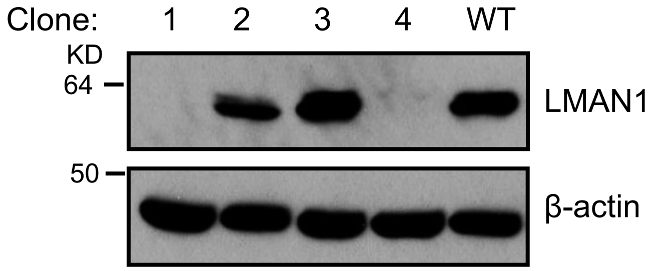

B

WT 5’-…GTGTTTGGATCAGCTGATCTGTGGAATGGTGTTGG…-3’

Allele-1 GTGTTTGGATCAGCT---------GAATGG (424-451del8bp)
Allele-2 GTGTTTGGATCAGCT---------GAATGG (424-451del8bp)

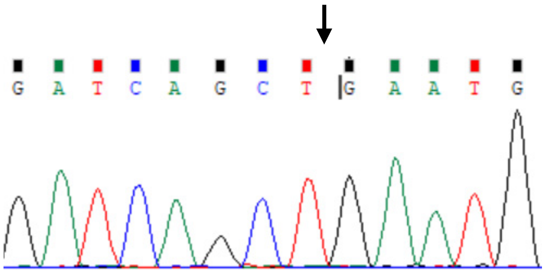

Fig. S1. Generation of LMAN1 KO HepG2 cells. (A) Immunoblotting of 4 clones revealed that clones 1 and 4 are KO cells with no LMAN1 expression. (B) Sequencing results showed that clone 1, which was used in subsequent experiments, is homozygous for an 8 bp deletion at the target site. Red letters denote the sequence for gRNA, and the protospacer adjacent motif (PAM) sequence is underlined. Upper case letters denote the sequence of *LMAN1* exon 3.

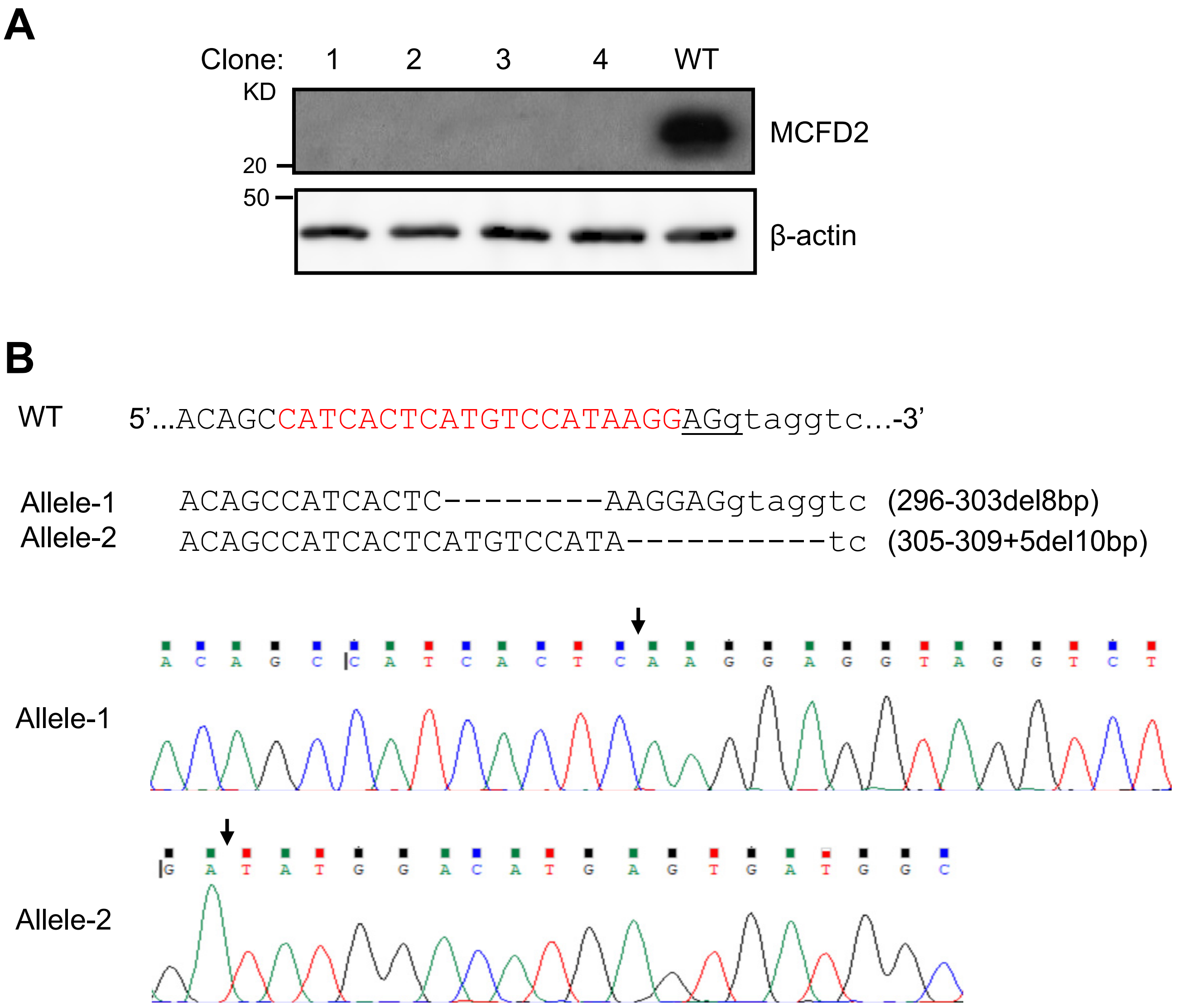

Fig. S2. Generation of MCFD2 KO HepG2 cells. (A) Immunoblotting of 4 clones revealed that all 4 clones are KO cells with no MCFD2 expression. (B) Sequencing results showed that clone 1, which was used in subsequent experiments, has an 8 bp deletion at one allele and a 10 bp deletion at the second allele. Red letters denote the sequence for gRNA, and the PAM sequence is underlined. Upper case letters denote *MCFD2* exon 3 sequence and lower case letters represent intron sequence.

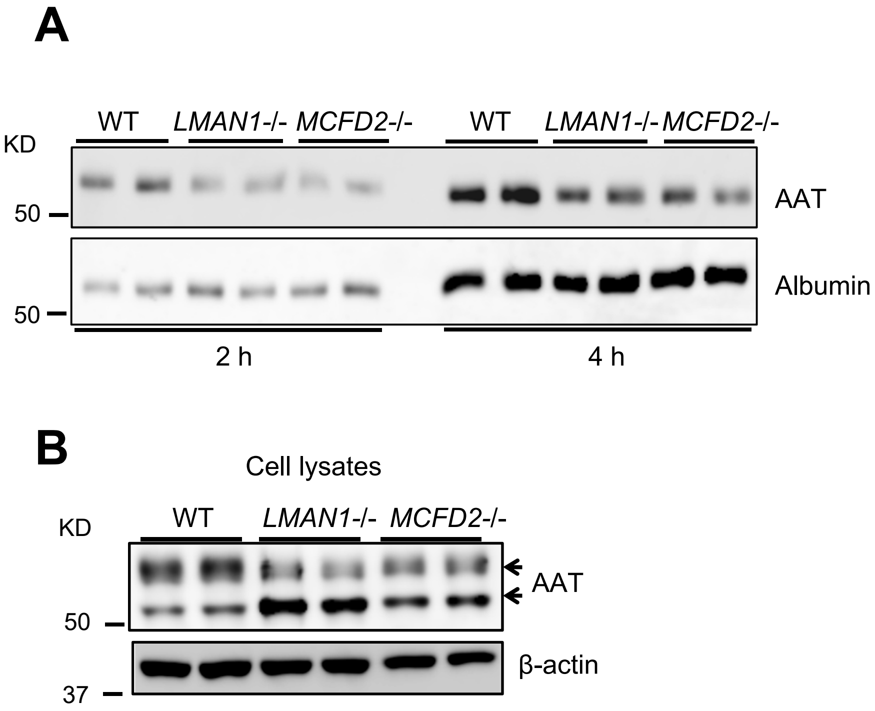

Fig. S3. Analysis of AAT secretion levels in LMAN1 and MCFD2 KO cells. (A) WT and two independent clones of LMAN1 and MCFD2 KO HepG2 cells were seeded in 6 well plates, and a medium change was carried out the next day. At 2 h and 4 h after the medium change, conditioned media were collected and subjected to immunoblotting with anti-AAT and anti-albumin antibodies. (B) Cells were collected 4 h after medium change. Cell lysates were analyzed by immunoblotting with anti-AAT and anti-β-actin antibodies. Arrows indicate mature (upper band, Golgi fraction) and immature (lower band, ER fraction) fractions in cell lysates.

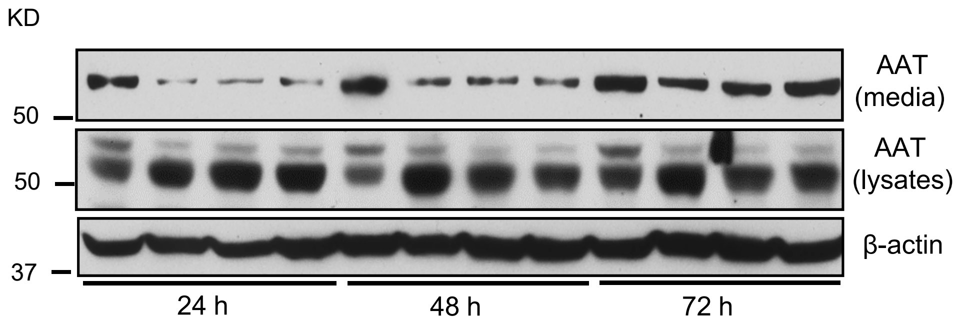

Fig. S4. Intracellular and secreted AAT in cultured primary hepatocytes. Hepatocytes were isolated from liver of WT, *Lman1*$^{-/-}$*, Mcfd2*$^{-/-}$ and *Lman1*$^{-/-}$*/Mcfd2*$^{-/-}$ (DKO) mice and cultured in serum free medium. At 24, 48 and 72 h after plating, conditioned media and cell lysates were analyzed by immunoblotting with an anti-AAT antibody. Cell lysates were also immunoblotted with anti-β-actin as loading controls.

Figure S4

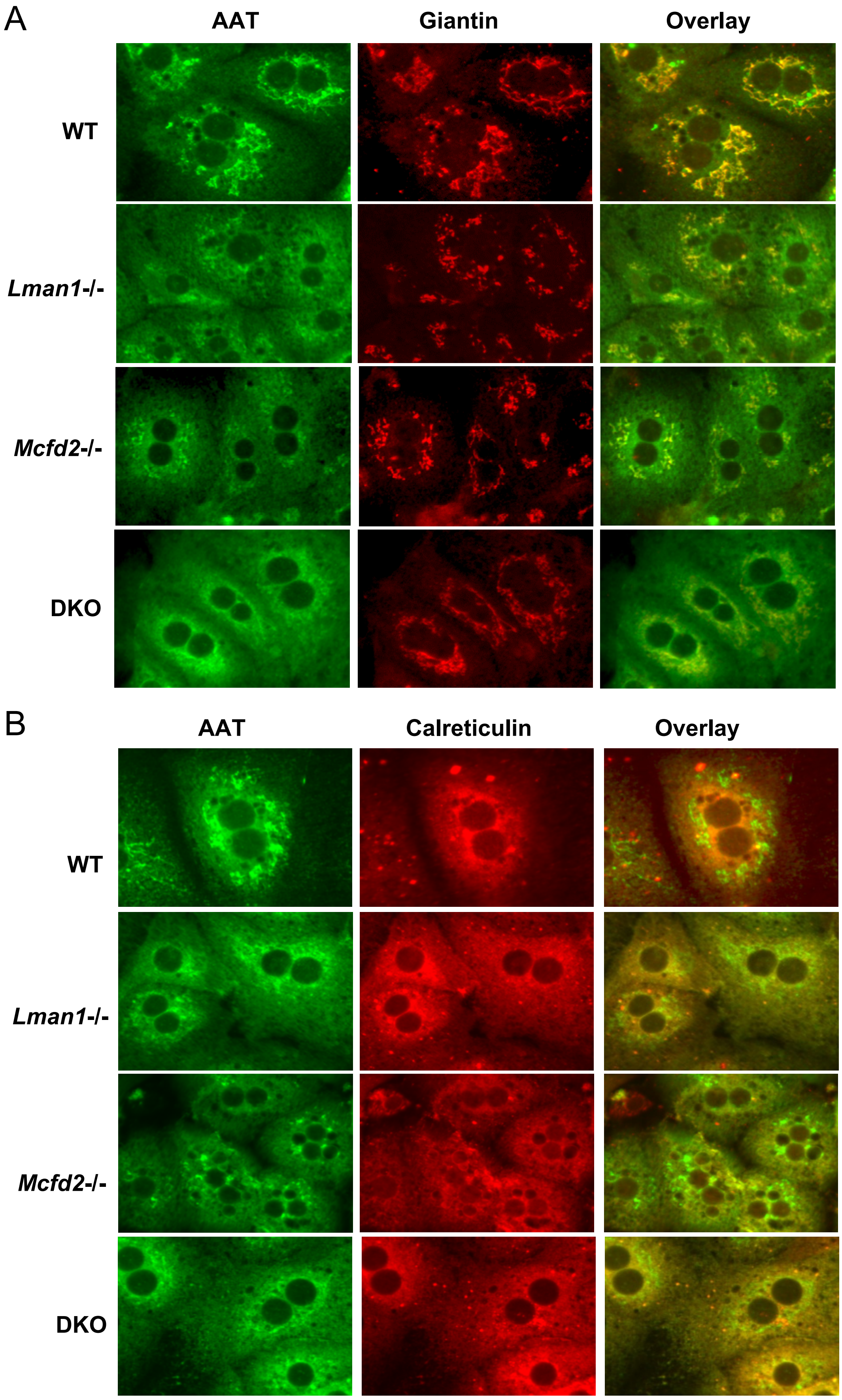
A
AAT
Giantin
Overlay
WT
Lman1-/-
Mcfd2-/-
DKO
B
AAT
Calreticulin
Overlay
WT
Lman1-/-
Mcfd2-/-
DKO

Figure S5

Fig. S5. Intracellular localization of AAT in primary hepatocytes from WT, *Lman1*-/-, *Mcfd2*-/- and DKO mice. (A) Immunofluorescence staining of hepatocytes cultured for 24 h with anti-mouse AAT and anti-giantin antibodies. (B) Immunofluorescence staining of hepatocytes cultured for 24 h with anti-mouse AAT and anti-calreticulin antibodies.

A

LMAN1 GTGTTTGGATCAGCTGATCTGTGGAATGGTGTTGG
exon 3

Allele-1 GTGTTTGGATCAGCTGATCTGT-GAATGG (451delG)
Allele-2 GTGTTTGGATCAGCTGATCTGT-GAATGG (451delG)
(293T)

B

MCFD2 ACAGCCATCACTCATGTCCATAAGGAGgtaggtctggcag
exon 3 *intron 3*

Allele-1 ACAGCCATCACTCATGT----AAGGAGgtaggtc (300-303delCCAT)
Allele-2 ACAGCCATCACTCATGTCCATA-GGAGgtaggtc (303delA)
(293T)

Fig. S6. Genotypes of CRISPR-Cas9 generated LMAN1 and MCFD2 KO HEK293T cells used in the study. (A) Sequencing results showed that the LMAN1 KO HEK293T cell clone has a homozygous 1 bp deletion. (B) Sequencing results showed that the MCFD2 KO HEK293T cell clone has a 4 bp deletion at one allele and a 1 bp deletion at the second allele. Red letters denote the sequence for gRNA, and the protospacer adjacent motif (PAM) sequence is underlined. Upper case letters denote exon sequences and lower case letters represent intron sequences.
